# Tubulin Alpha 1b Is Associated with the Immune Cell Infiltration and the Response of HCC Patients to Immunotherapy

**DOI:** 10.3390/diagnostics12040858

**Published:** 2022-03-30

**Authors:** Xinyao Hu, Hua Zhu, Biao Chen, Xiaoqin He, Yang Shen, Xiaoyu Zhang, Wenliang Chen, Xin Liu, Yangtao Xu, Ximing Xu

**Affiliations:** 1 Cancer Center, Renmin Hospital of Wuhan University, Wuhan University, Wuhan 430060, China; 2016302180225@whu.edu.cn (X.H.); cb2022@yeah.net (B.C.); whuhexq@aliyun.com (X.H.); 2017302180196@whu.edu.cn (Y.S.); 2019203020063@whu.edu.cn (X.Z.); chenwenliang11@126.com (W.C.); yvome@foxmail.com (X.L.); 2015302180193@whu.edu.cn (Y.X.); 2 Department of Neurosurgery, Renmin Hospital of Wuhan University, Wuhan University, Wuhan 430060, China; zhuhua0411@whu.edu.cn

**Keywords:** TUBA1B, HCC, immunotherapy, immune infiltration cells, prognosis

## Abstract

Tubulin alpha 1b (TUBA1B) is an important microtubule isoform that is involved in the formation of the cytoskeleton. The objective of our study was to explore the potential of TUBA1B in predicting the prognosis of HCC and patients’ response to immunotherapy. Raw data was extracted from TCGA and GEO databases, and then HCCDB, TIMER, HPA, and GEPIA websites, as well as R software, were used to perform bioinformatics analysis to investigate the potential of TUBA1B as a prognostic and immunotherapeutic marker for hepatocellular carcinoma (HCC). We found that both TUBA1B mRNA and protein were highly expressed in HCC. TUBA1B was proved to be an independent prognostic predictor of HCC. Additionally, TUBA1B expression was associated with the infiltration of several immune cells in HCC. Moreover, TUBA1B was coexpressed with immune-related genes and immune checkpoints. Patients expressing high TUBA1B responded better to immune checkpoint inhibitor (ICI) therapy. GO and KEGG analyses revealed that TUBA1B may be involved in the processes of cell cycle, spliceosome, and DNA replication. In conclusion, TUBA1B is expected to be a prognostic and immunotherapeutic marker for HCC.

## 1. Introduction

Primary liver cancer, which accounts for 6% of all cancers and 9% of all death from cancer worldwide, is a cancer in urgent need of further research. Hepatocellular carcinoma (HCC) accounts for about three quarters of primary liver cancers [1,2,3]. The complex etiologic factors and high level of heterogeneity make the diagnosis and prognosis of HCC challenging [4]. Furthermore, although traditional surgical resection or liver transplantation can be used to treat HCC, the chances of recurrence are high as HCC is often at an advanced stage when detected [2]. Therefore, it is urgent to discover new diagnostic and prognostic markers.

Tubulin is an essential cytoskeletal component that comes in five different forms, α-, β-, γ-, δ-, and ε-tubulin. α- and β-tubulin heterodimers constitute microtubules, which participate in cell motility, adhesion, generation, and division. Tubulin alpha 1b (TUBA1B) is an important isoform of α-tubulin [5,6]. It has been reported that high TUBA1B expression is correlated with adverse outcome and resistance to the paclitaxel of HCC patients [7]. DNA hypomethylation of TUBA1B was observed in HCC tissues [8]. Moreover, TUBA1B was reported to be upregulated in Wilms tumor [9]. Moreover, it was revealed that TUBA1B expression was related to the overall survival (OS) of colon adenocarcinoma (COAD) patients and contributes to the exhaustion of the CD8^+^ T cell [10]. In addition, TUBA1B was regarded as a key regulator of osteosarcoma [11]. A model containing TUBA1B was created to differentiate pancreatic ductal adenocarcinoma patients and worked well [12]. Overall, the role of TUBA1B in cancers is receiving increasing attention and deserves to be explored in depth to further investigate its action mechanism and potential as a prognostic marker.

It is well known that tumor-infiltrating immune cells (TIICs) are essential components of the tumor microenvironment (TME) and that they are closely linked to the development of tumors [13]. Recently, a variety of potent chemotherapy and radiotherapy have been employed to reinstate immune monitoring by activating the immune response [14]. Immunotherapy has received increasing attention in the treatment of cancers, with cytokine and immune checkpoint inhibitor (ICI) therapies becoming therapeutic strategies for various types of cancer [15]. It is therefore of interest to tap into biomarkers that predict the efficacy of immunotherapy so as to screen out patients suitable for immunotherapy. However, the relationship of TUBA1B expression with immune cell infiltration in HCC has not been investigated. No studies have examined whether TUBA1B can be used as a biomarker to guide immunotherapy in HCC.

In our study, we first evaluated the differential expression of TUBA1B in HCC tissues and normal tissues. Next, we comprehensively assessed the relationship between TUBA1B expression and the clinicopathology and prognosis of HCC using multiple bioinformatic approaches. We also analyzed the correlation between TUBA1B and TME, TIIC, immune checkpoints, and immune-related genes. The protein–protein interaction (PPI) information of TUBA1B was detected, and the potential functions of TUBA1B were explored by gene enrichment analysis. Our findings provide novel perspectives on the role of TUBA1B and offer new targets for the diagnosis, therapy, and prognosis of HCC.

## 2. Methods

### 2.1. Data Collection and Differential Expression Analysis

Cancer-related RNA sequences, survival data, and clinicopathology for HCC were obtained from the UCSC Xena website (https://xena.ucsc.edu/, originally from TCGA) (accessed on 14 January 2022) website. TUBA1B expression data were downloaded from the TCGA (https://tcga.xenahubs.net) (accessed on 14 January 2022) database and processed and analyzed by Perl and R software. We used the “Wilcox.test” method to assess the differential mRNA expression of TUBA1B in HCC and normal tissues. False discovery rate (FDR) values < 0.05 were used as cut-off values. The boxplot was plotted with the “ggpubr” R package. Moreover, we also extracted the expression dataset from the GEO (http://www.ncbi.nlm.nih.gov/geo/) (accessed on 14 January 2022) [16], including GSE46408 (6 HCC samples, 6 normal samples) and GSE84402 (14 HCC samples, 12 normal samples). The boxplot were then visualized using the “ggpubr” package in R software. To ensure the authenticity of the results, we used gene expression profiling interactive analysis (GEPIA) (http://gepia.cancer-pku.cn/index.html) (accessed on 15 January 2022), a website tool for normal and cancer gene expression profiling and interactive analyses using data from TCGA and GTEx [17], to further validate our results. HCCDB (http://lifeome.net/database/hccdb.html) (accessed on 15 January 2022), an online resource dedicated to assisting in the exploration of HCC gene expression [18], and TIMER (https://cistrome.shinyapps.io/timer/) (accessed on 17 January 2022), a database for TIICs analysis [19], were used to further verify the differential *TUBA1B* expression between normal and HCC tissues.

Immunohistochemical images of the TUBA1B protein expression in HCC and normal tissues were investigated immediately afterward to assess the differential TUBA1B protein expression in the human protein atlas (HPA, https://www.proteinatlas.org/) database (accessed on 19 January 2022).

### 2.2. The Relationship of Survival and Clinicopathology with TUBA1B in HCC

Univariate and multivariate Cox regression analyses were performed to identify the proper items for creating column line plots. The *p*-value, 95% CI and HR for each term, were displayed via the R software using the “forestplot” R package. Our study assessed TUBA1B and main prognostic and clinical factors including grade, age, gender, radiotherapy, and race. Nomograms were developed using R software and the “rms” package to predict total recurrence at 1, 3, and 5 years according to the findings of the multivariate Cox proportional hazards analysis.

The survival information was obtained from the TCGA database. The OS, disease-specific survival (DSS), progression-free survival (PFS), and disease-free survival (DFS) were employed to clarify the association of TUBA1B and the HCC patients’ prognosis. We used Kaplan-Meier (KM) and log-rank tests to perform survival analysis, with survival curves plotted using the R packages “survivor” and “survminer”. Cox analysis was carried out using the “survival” package, and clinicopathological correlations were conducted using the R packages “ggpubr” and “limma”.

### 2.3. Analysis of Immune Infiltrates, Immune Checkpoint, and Immune-Related Genes

The TIMER database was used to investigate the correlation of TUBA1B with TIIC, including neutrophils, B cells, dendritic cells, CD4^+^ T cells, macrophages, and CD8^+^ T cells in HCC. Additionally, the R packages “limma” and “estimate” were used to calculate stromal and immune scores using the ESTIMATE algorithm, a method that uses gene expression signatures to infer the fraction of stromal and immune cells in tumor samples [20]. Based on TUBA1B expression data, we analyzed tumor purity and TIICs in HCC tissues using CIBERSORT, a method to characterize the cell composition of tissues from their gene expression profiles [21]. We used the R packages “ggplot2”, “ggpubr”, and “ggExtra” to analyze the relationship of TUBA1B with TME or immune cell infiltration.

Subsequently, we selected PDCD1, CTLA4, SIGLEC15, HAVCR2, TIGIT, CD274, PDCD1LG2, and LAG3 as immune checkpoint-associated transcripts and extracted their expression data. In addition, we analyzed the expression data of chemokines, chemokine receptors, major histocompatibility complexes (MHC), and immunoinhibitory and immunostimulatory genes. R packages “ggplot2”, “immuneeconv”, “pheatmap”, “limma”, ”RColorBrewer”, and “reshape2” were used to evaluate the coexpression of TUBA1B with these genes. Moreover, we applied the tumor immune dysfunction and exclusion (TIDE) algorithm to evaluate the patients’ response to ICI therapy.

### 2.4. Conduction of Protein Protein Interaction (PPI) Network

The TUBA1B PPI information were constructed by GeneMANIA (http://genemania.org/search/) (accessed on 2 February 2022) [22] and STRING (https://cn.string-db.org/) (accessed on 2 February 2022) websites to further investigate the functions and mechanisms of TUBA1B.

### 2.5. TUBA1B Enrichment Analyses in HCC

GO functional annotation and KEGG enrichment pathway analysis of TUBA1B were performed in LinkedOmics (http://www.linkedomics.org/) (accessed on 3 February 2022), a web server for analyzing multi-omics data in 32 types of cancer [23].

### 2.6. Statistical Analysis

The statistics of our research were analyzed using the R software (version 4.0.2). Gene expression information was processed for normalization by log2 transformation. The cut off was median expression to define the high or low expression. Tumor and normal tissues were compared using a two-group *t*-test. Groups ≥ 3 were compared by Kruskal-Wallis one-way ANOVA. All survival analyses were conducted using the KM analysis, log-rank test and Cox proportional hazards model. Pearson’s test or Spearman’s test was used to analyze the association of two variables. *p*-value < 0.05 was regarded as significant.

## 3. Results

### 3.1. Differential Expression of TUBA1B in Normal and HCC Tissues

**Figure 1:**
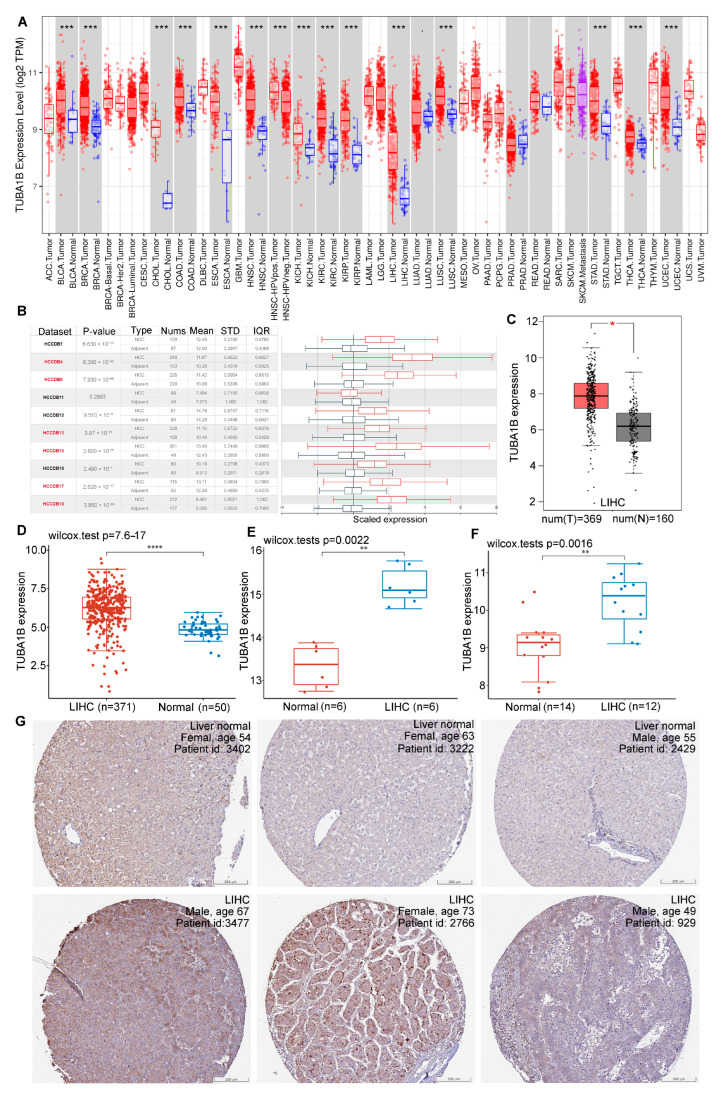
Differential expression of TUBA1B in normal and HCC tissues. (**A**) TUBA1B expression in 33 types of cancers in TIMER. (**B**) TUBA1B expression levels in 1010 LIHC cohorts in HCCDB. (**C**) Different TUBA1B expression in normal and LIHC tissues in GEPIA. (**D**) Different TUBA1B expression in LIHC and normal tissues in TCGA. (**E**,**F**) Different TUBA1B expression in LIHC and normal tissues in GEO. (**G**) IHC staining of TUBA1B in liver normal tissues (above) and LIHC tissues (below). * *p* < 0.05, ** *p* < 0.01, *** *p* < 0.0001, **** *p* < 0.00001.

To obtain convincing results, we investigated differential TUBA1B mRNA expression in normal and HCC tissues using three databases and R software. TIMER online tool revealed that TUBA1B was upregulated in liver hepatocellular carcinoma (LIHC) (*p* < 0.001) (Figure 1A). We then found the same trends in 10 LIHC cohorts in the HCCDB (Figure 1B). Higher expression of TUBA1B was also observed in LIHC than in normal tissues in GEPIA (Figure 1C). Additionally, we handled the data from TCGA with R software and found that TUBA1B was significantly upregulated in LIHC (*p* < 0.0001) (Figure 1D). Ultimately, the GSE46408 (Figure 1E, *p* = 0.0022) and GSE84402 (Figure 1F, *p* = 0.0016) datasets from GEO also revealed that TUBA1B was significantly overexpressed in HCC.

The immunohistochemical images of TUBA1B in HCC from HPA also confirmed that TUBA1B protein was also upregulated in HCC tissues. Immunohistochemistry (IHC) staining was weaker in normal tissues than in LIHC tissues (Figure 1G).

### 3.2. Relationship of TUBA1B Expression with OS, DFS, PFS, and DSS

**Figure 2:**
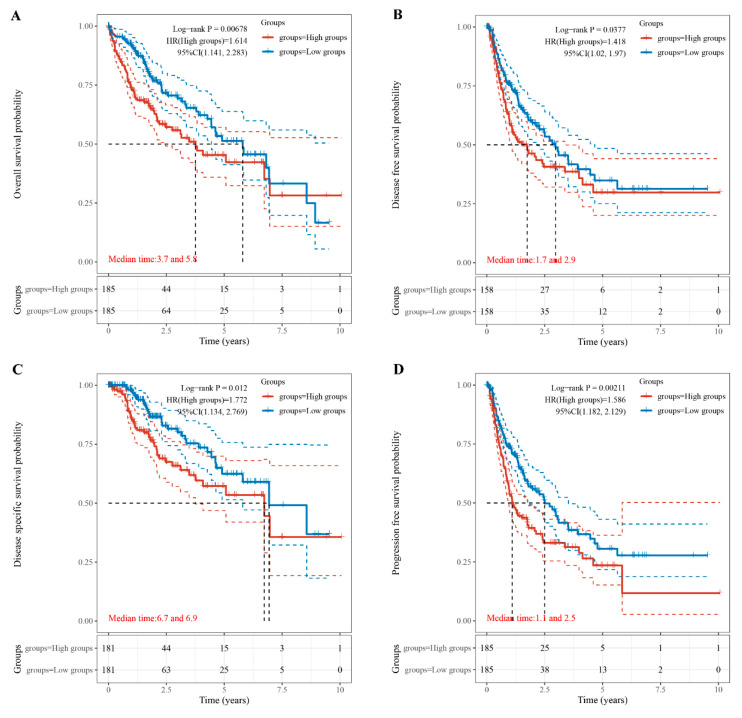
Relationship of TUBA1B expression with OS, DFS, DSS, and PFS. (**A**) High TUBA1B expression groups had a shorter OS. (**B**) High TUBA1B expression groups had a shorter DFS. (**C**) High TUBA1B expression groups had a shorter DSS. (**D**) High TUBA1B expression groups had a shorter PFS. The upper and lower dashed lines are 95% confidence intervals (CI).

The correlation of TUBA1B expression with OS, DFS, DSS, and PFI was further investigated to explore the potential of TUBA1B as a prognostic marker for HCC. KM survival curves showed that high TUBA1B expression led to a shorter OS in patients with HCC (Figure 2A, *p* = 0.00678). DFS was also distinctly shorter in the high TUBA1B expression group than the group of low TUBA1B expression (Figure 2B, *p* = 0.0377). Moreover, HCC patients with low TUBA1B expression had longer DSS (Figure 2C, *p* = 0.012) and PFS (Figure 2D, *p* = 0.00211). Overall, high TUBA1B expression predicts a poor prognosis for HCC patients.

### 3.3. TUBA1B Correlated with Clinicopathology and Its Prognostic Potential

**Figure 3:**
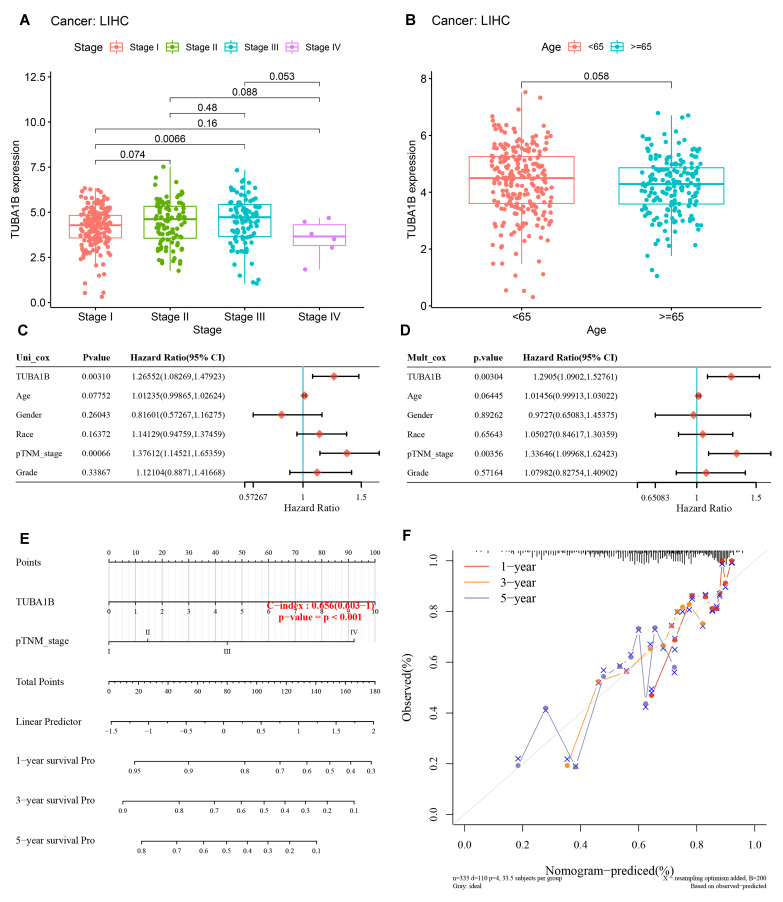
The relationship of clinicopathology with TUBA1B in HCC and the prognostic value of TUBA1B. (**A**) TUBA1B expression in different stages of HCC. (**B**) TUBA1B expression in age < 65 and age ≥ 65 of HCC. The univariate (**C**) and multivariate (**D**) Cox analysis of TUBA1B, age, gender, race, pTNM_stage, and grade. (**E**) The nomogram of TUBA1B and pTNM_stage was constructed to predict 1-, 3-, and 5-year OS in LIHC patients. (**F**) Calibration curves of the nomogram. Circles and cross are the actual observed mortality rates and 95% CI.

We further investigated the relationship of TUBA1B expression with tumor stage and age. We found TUBA1B was more overexpressed in stage Ⅲ than in stage Ⅰ of LIHC (*p* = 0.0066) (Figure 3A). Notably, the expression of TUBA1B in stage IV of LIHC was not significantly higher than in stage I, which may be related to the insufficient case sample. Moreover, no apparent relationship between TUBA1B expression and age of LIHC patients was detected (Figure 3B). Next, we conducted univariable and multivariable Cox regression analyses. The univariate Cox regression model indicated that TUBA1B [hazard ratio (HR) = 1.26552, *p* = 0.00310], pTNM_stage (HR = 1.37612, *p* = 0.00066) (Figure 3C) were independent prognostic factors for HCC. Similarly, in the multivariable regression model, TUBA1B (HR = 1.2905, *p* = 0.00304) (Figure 3D) and pTNM_stage (HR = 1.33646, *p* = 0.00356) (Figure 3D) also had prognostic values. We constructed a nomogram that combined two independent prognostic factors, comprising pTNM_stage and TUBA1B, to predict 1-, 3-, and 5-year OS in patients with LIHC (Figure 3E). The higher the total score obtained by summing the patient’s scores for the two prognostic factors, the worse the patient’s outcome. Furthermore, the calibration curve revealed that the nomogram performed well in estimating OS at 1, 3, and 5 years (Figure 3F). Taken together, TUBA1B has potential as a prognostic factor for HCC.

### 3.4. TUBA1B Expression Was Related to Immune Cell Infiltration in LIHC

**Figure 4:**
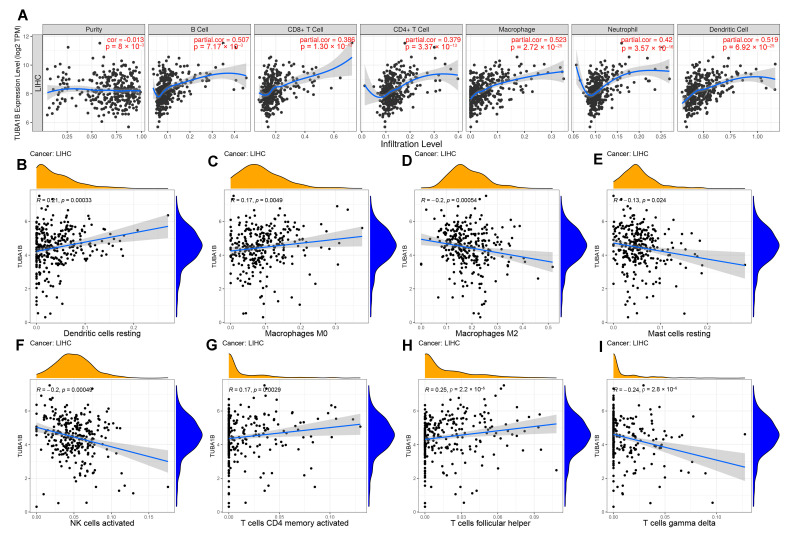
TUBA1B expression was in relation to immune cell infiltration in LIHC. (**A**) The relationship of TUBA1B expression with tumor purity and the infiltration of major immune cells including CD4+ T cells, B cells, CD8+ T cells, macrophage, dendritic cell, and neutrophil from TIMER. The relationship of TUBA1B expression with resting dendritic cells (**B**), macrophages M0 (**C**), macrophages M2 (**D**), resting mast cells (**E**), activated NK cells (**F**), activated memory CD4 T cells (**G**), T cells follicular helper (**H**), and T cells gamma delta (**I**) explored using the CIBERSORT algorithm.

TIICs are important components of TME and have a significant influence on cancer progression. Hence, we first explored the relationship of TUBA1B with tumor purity and major TIICs. The results in the TIMER database showed that high TUBA1B expression promoted the infiltration of B cells (*r* = 0.507, *p* < 0.001), CD4+ T cells (*r* = 0.379, *p* < 0.001), CD8+ T cells (*r* = 0.386, *p* < 0.001), neutrophils (*r* = 0.42, *p* < 0.001), dendritic cells (*r* = 0.519, *p* < 0.001), and macrophages (*r* = 0.523, *p* < 0.001) (Figure 4A). Next, we used the CIBERSORT algorithm to further evaluate the correlation between TIIC infiltration and TUBA1B expression in LIHC. We found TUBA1B expression was positively related to the infiltration of resting dendritic cells (Figure 4B, *r* = 0.21, *p* = 0.00033), macrophages M0 (Figure 4C, *r* = 0.17, *p* = 0.0049), activated memory CD4 T cells (Figure 4G, *r* = 0.17, *p* = 0.0029), and T cells follicular helper (Figure 4H, *r* = 0.25, *p* = 2.2 × 10^−5^). In contrast, macrophages M2 (Figure 4D, *r* = −0.2, *p* = 0.00054), resting mast cells (Figure 4E, *r* = −0.13, *p* = 0.024), activated natural killer (NK) cells (Figure 4F, *r* = −0.2, *p* = 0.00049), and T cells gamma delta (Figure 4G, *r* = 0.17, *p* = 0.0029) infiltration was negatively associated with TUBA1B expression.

### 3.5. Correlation of TUBA1B Expression with Immune Checkpoints, Immune-Related Genes, and Patient Response to Immunotherapy

**Figure 5:**
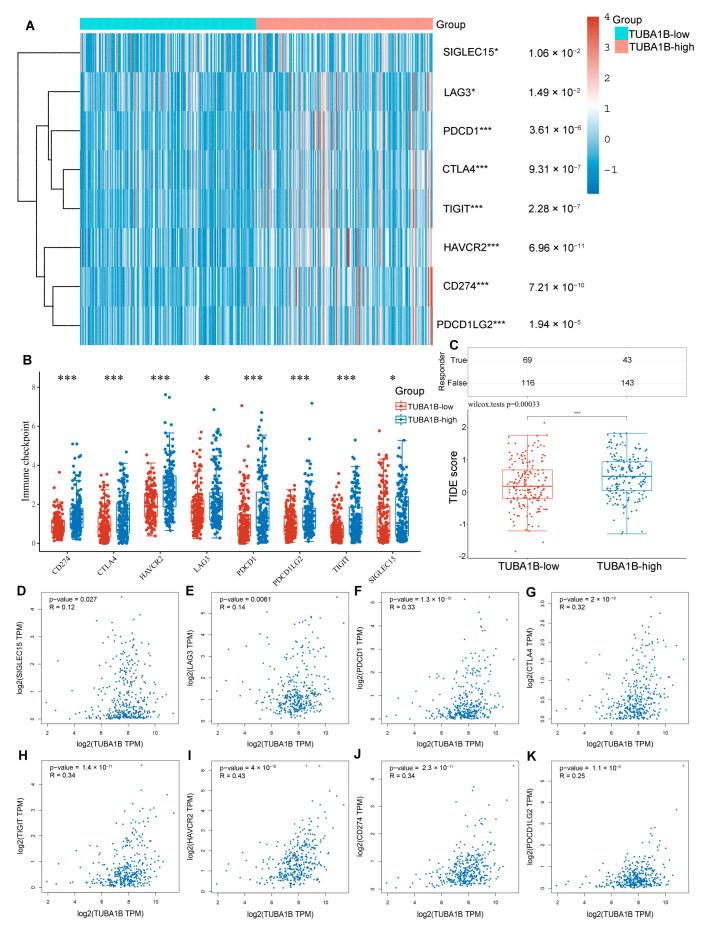
The relationship between TUBA1B expression and immune checkpoints and patients’ response to immunotherapy. (**A**) Heat map of the correlation between immune checkpoint genes expression and TUBA1B expression in HCC. (**B**) Histogram of the association between immune checkpoint genes expression and TUBA1B expression in HCC. (**C**) Relationship between TIDE scores and TUBA1B expression in HCC. The relationship of TUBA1B expression with the expression of SGLEC15 (**D**), LAG3 (**E**), PDCD1 (**F**), CTLA4 (**G**), TIGIT (**H**), HAVCR2 (**I**), CD274 (**J**), and PDCD1LG2 (**K**). * *p* < 0.01, *** *p* < 0.001.

Subsequently, we explored the relationship between TUBA1B expression and immune checkpoints. We found that a high expression of TUBA1B was associated with high immune checkpoint genes expression (Figure 5A,B), including SIGLEC15 (*p* = 1.06 × 10^−2^), LAG3 (*p* = 1.49 × 10^−2^), PDCD1 (*p* = 3.61 × 10^−6^), CTLA4 (*p* = 9.31 × 10^−7^), TIGIT (*p* = 2.28 × 10^−7^), HAVCR2 (*p* = 6.96 × 10^−11^), CD274 (*p* = 7.21 × 10^−10^), and PDCD1LG2 (*p* = 1.94 × 10^−5^). In addition, we used the GEPIA 2.0 database to analyze the relationship of TUBA1B expression with these immune checkpoint genes in LIHC and observed that TUBA1B expression was positively correlated with the expression of SIGLEC15 (Figure 5D, *r* = 0.12, *p* = 0.027), LAG3 (Figure 5E, *r* = 0.14, *p* = 0.0061), PDCD1 (Figure 5F, *r* = 0.33, *p* = 1.3 × 10^−10^), CTAL4 (Figure 5G, *r* = 0.32, *p* = 2 × 10^−10^), TIGIT (Figure 5H, *r* = 0.34, *p* = 1.4 × 10^−11^), HAVCR2 (Figure 5I, *r* = 0.43, *p* = 4 × 10^−18^), CD274 (Figure 5J, *r* = 0.34, *p* = 2.3 × 10^−11^), and PDCD1LG2 (Figure 5K, *r* = 0.25, *p* = 1.1 × 10^−6^). Additionally, we explored the relationship of TUBA1B expression with the intensity of response to immunotherapy in HCC patients. The results indicated that the TUBA1B high expression group had a higher score of TIDE, which predicted a better response to immunotherapy (Figure 5C, *p* = 0.00033).

**Figure 6:**
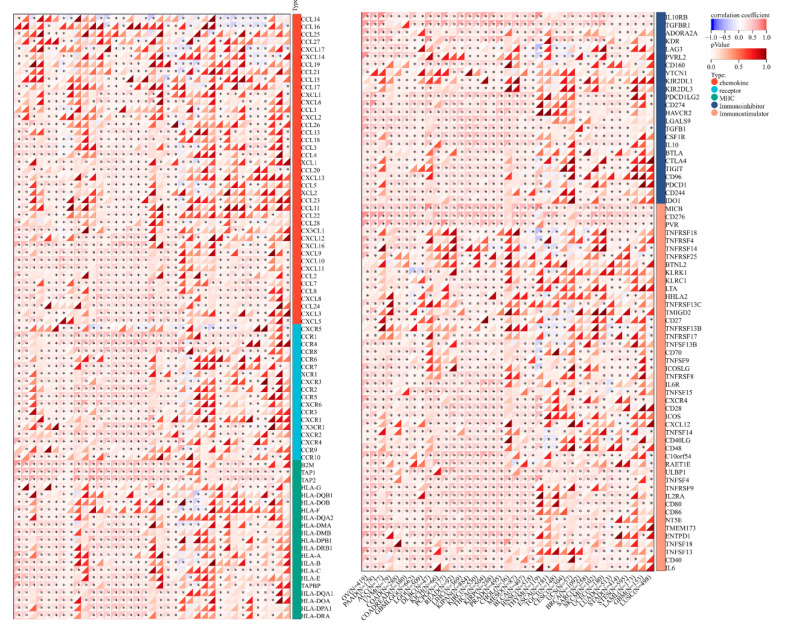
Pan-cancer analysis of the correlation between TUBA1B expression and immune-related genes. * *p* < 0.05.

The correlation between TUBA1B and immune-related genes expression was then explored. The results showed that TUBA1B was coexpressed with almost all chemokine, chemokine receptor, MHC, and immunoinhibitory and immunostimulatory genes in HCC. TUBA1B was also highly correlated with immune-related genes in many other cancers, including pancreatic adenocarcinoma (PAAD), ovarian serous cystadenocarcinoma (OV), thyroid carcinoma (THCA), kidney renal clear cell carcinoma (KIPAN + KIRC + KIRP), and prostate adenocarcinoma (PRAD), suggesting that TUBA1B may affect the immune microenvironment in these cancers (Figure 6).

### 3.6. Construction of PPI Networks for TUBA1B, Analysis of GO Function Annotation, and KEGG Pathway Enrichment of TUBA1B

**Figure 7:**
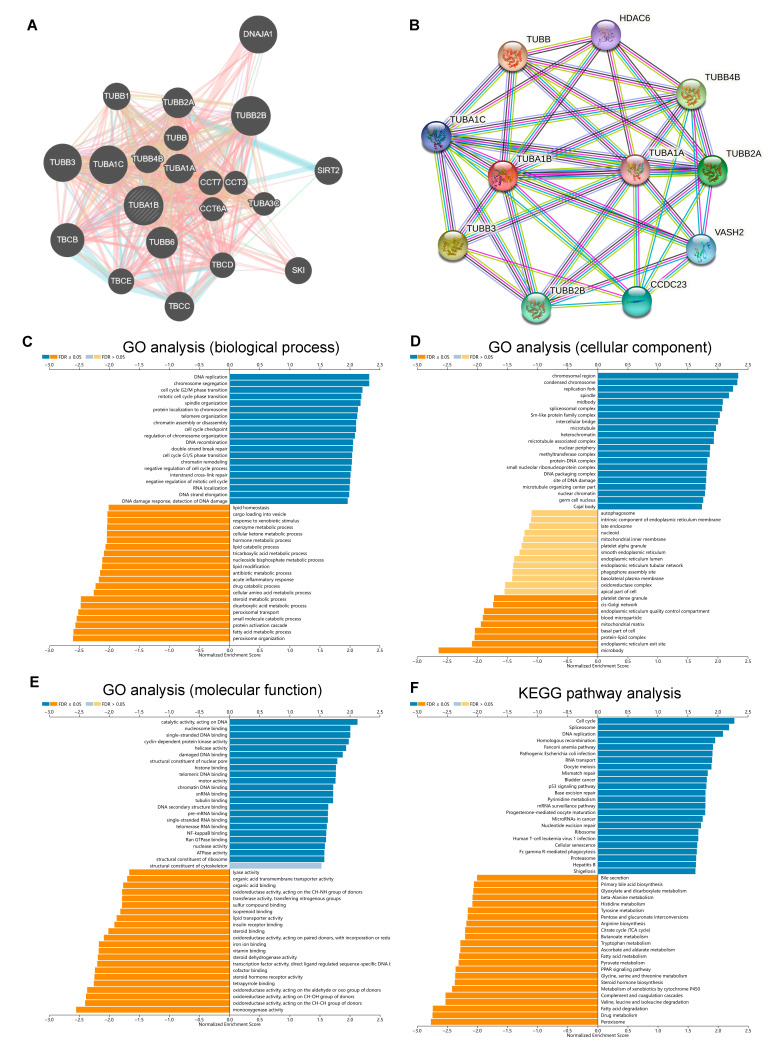
Construction of PPI networks for TUBA1B and analysis of GO function annotation and KEGG pathway enrichment of TUBA1B. (**A**) PPI network of TUBA1B constructed from GeneMANIA. (**B**) PPI network of TUBA1B constructed from STRING. (**C**) GO analysis of the biological process of TUBA1B. (**D**) GO analysis of cellular component of TUBA1B. (**E**) GO analysis of molecular function of TUBA1B. (**F**) KEGG pathway analysis of TUBA1B.

We used GeneMANIA and STRING databases to construct PPI networks for TUBA1B. The PPI network from GeneMANIA indicated that TUBA1B was coexpressed with CCT6A, CCT3, CCT7, TUBA1A, TUBB4B, TUBA1B, TUBB6, SIRT2, DNAJA1, and so on (Figure 7A). Moreover, the PPI network from STRING revealed that TUBA1B was coexpressed with TUBA1B, TUBB3, TUBB2B, CCDC23, VASH2, TUBA1A, HDAC6, TUBB2A, TUBB4B, and TUBB (Figure 7B).

Next, we predicted the potential function of TUBA1B in HCC using the LinkedOmics database. GO analysis of biological process (Figure 7C) indicated that TUBA1B was positively correlated with the process of DNA replication, cell cycle G2/M phase transition, chromosome segregation, mitotic cell cycle phase transition, spindle organization, protein localization to chromosome, etc. In addition, TUBA1B was negatively correlated with the process of peroxisome organization, fatty acid metabolic process, protein-activated cascade, small molecule catabolic process, peroxisomal transport, etc. Moreover, GO analysis of the cellular component indicated genes correlated with TUBA1B were mainly located in chromosomal region, condensed chromosome, replication fork, spindle and midbody (Figure 7D). GO analysis of molecular function (Figure 7E) revealed that TUBA1B may work through catalytic activity, single-stranded DNA binding, nucleosome binding, acting on DNA, cyclin-dependent protein kinase activity, helicase activity, etc. KEGG pathway analysis (Figure 7F) also indicated that TUBA1B was positively correlated with the process of cell cycle, spliceosome, homologous recombination, DNA replication, Fanconi anemia pathway, etc., and was negatively associated with the process of peroxisome, drug metabolism, valine, leucine and isoleucine degradation, fatty acid degradation, etc.

## 4. Discussion

As the most common fatal malignancy worldwide and the only one of the top five deadliest cancers that increases in percentage each year [24], liver cancer deserves more in-depth research to find valuable prognostic markers and therapeutic targets. Traditional advanced HCC treatment drugs, such as sorafenib and lenvatinib, have limited efficacy and considerable toxicity [25]. The emerging ICI immunotherapy has been shown to produce meaningful improvement in clinical outcomes in HCC, but its objective response rate as a monotherapy for HCC is only 15–20% [26]. Thus, it is urgent to define biomarkers to differentiate immunotherapy-sensitive patients from nonsensitive patients, so that appropriate combination therapy can be administered. TUBA1B, a prominent isoform of microtubule, is an important component of the cytoskeleton and is engaged in various vital biological processes. It has proved to be upregulated in various cancers. Our study is dedicated to exploring the feasibility of TUBA1B as a prognostic and immunotherapy marker for HCC patients.

We first investigated the differential TUBA1B expression in normal and HCC tissues. We found that both mRNA and protein of TUBA1B were notably overexpressed in HCC. Next, we explored the correlation between TUBA1B and the prognosis of HCC, and observed high probability of OS, DFS, DSS, and PFS in HCC in TUBA1B-low groups, indicating that high TUBA1B expression predicted a poor prognosis in HCC. Moreover, the role of TUBA1B in the clinicopathology of HCC was confirmed. The results show that TUBA1B was notably higher in stage Ⅲ than stage Ⅰ in HCC. Additionally, the univariate and multivariate Cox analysis demonstrated that TUBA1B was an independent prognostic factor for LIHC. We further constructed a nomogram using TUBA1B and pTNM_stage and achieved good results in predicting 1-, 3-, and 5-year OS in HCC patients. Overall, TUBA1B was a potential prognostic biomarker for HCC.

Subsequently, our research explored the role of TUBA1B in tumor immunity for the first time. The findings showed that TUBA1B was closely correlated with TIIC in HCC and was positively related to infiltration of B cells, CD4^+^ T cells, CD8^+^ T cells, macrophages, dendritic cells, and neutrophils. We further analyzed the effect of TUBA1B on different immune cell subtypes and found that TUBA1B has different effects on different subtypes of the same cell type. Various published reports have declared that CD8^+^ T cells and CD4^+^ T cells play an opposing pro and anticancer role, and their effects are suppressed by Treg cells. The role of B cells in HCC remains controversial [27]. Macrophage M1 acts as a proinflammatory and antitumor agent whereas macrophage M2 acts as an anti-inflammatory and protumor agent, and macrophage polarization influences the development of cancers [28]. Moreover, neutrophils release neutrophil extracellular traps that suppress the immune response and promote cancer cell growth [29]. Dendritic cells can inhibit tumor growth [30]. The mechanism of TUBA1B regulation of these TIICs is complex and needs further research, but the oncogenic effect is more prominent ultimately.

Next, we investigated the correlation of TUBA1B expression with immune checkpoints, immune-related genes, and patients’ response to immunotherapy. The results show that TUBA1B was positively associated with various immune checkpoint genes expression, including SIGLEC15, LAG3, PDCD1, HAVCR2, TIGIT, CTLA4, PDCD1LG2, and CD274. Because overexpression of immune checkpoint genes is correlated with T-cell depletion and poor outcome of patients, this partly explains the carcinogenic role of TUBA1B. We also found that the TUBA1B high expression group had a higher score of TIDE, suggesting that outcomes of ICI treatment are better in patients with high TUBA1B expression. Moreover, TUBA1B was coexpressed with almost all chemokine, chemokine receptor, MHC, immunoinhibitory and immunostimulatory genes in LIHC, and it was also highly correlated with immune-related genes in many other cancers. Overall, TUBA1B has an essential role in cancer immunity.

Finally, we investigated genes and pathways associated with TUBA1B to further explore the underlying role of TUBA1B. We found that TUBA1B was coexpressed with genes of the CCT gene family and genes of tubulin alpha and tubulin beta. As CCT genes play a prominent role in the folding of many important proteins during cell division, it is emerging as a key molecule in mitosis [31]. Tubulin alpha and tubulin beta heterodimers form microtubules that are key cytoskeletal elements of all eukaryotic cells [32]. Taken together, TUBA1B may act through regulation of the cytoskeleton and mitosis. GO analysis revealed that TUBA1B was mainly located in the chromosomal region and was positively correlated with the process of DNA replication, cell cycle G2/M phase transition, chromosome segregation, mitotic cell cycle phase transition, etc. KEGG pathway analysis also showed that TUBA1B may be involved in cell DNA replication, cycle, spliceosome, and other processes. This is consistent with the conclusions drawn from our analysis of coexpressed genes of TUBA1B.

## 5. Conclusions

In conclusion, our study confirms that TUBA1B expression is elevated in HCC and high TUBA1B expression is correlated with a poor outcome in patients with HCC. In addition, TUBA1B is associated with immune cell infiltration, immune checkpoint genes expression, and immune-related genes expression in HCC. Patients with high TUBA1B expression responded better to ICI treatment. Additionally, TUBA1B may act through the regulation of the cytoskeleton as well as mitosis, and it is involved in a variety of important pathways. TUBA1B has great potential as a prognostic and immunotherapeutic marker for HCC.

## Data Availability

The datasets presented in this study can be found in online repositories. The names of the repository/repositories and accession number(s) can be found in the article.
